# Mutations in *CEP120* cause Joubert syndrome as well as complex ciliopathy phenotypes

**DOI:** 10.1136/jmedgenet-2016-103832

**Published:** 2016-05-06

**Authors:** Susanne Roosing, Marta Romani, Mala Isrie, Rasim Ozgur Rosti, Alessia Micalizzi, Damir Musaev, Tommaso Mazza, Lihadh Al-gazali, Umut Altunoglu, Eugen Boltshauser, Stefano D'Arrigo, Bart De Keersmaecker, Hülya Kayserili, Sarah Brandenberger, Ichraf Kraoua, Paul R Mark, Trudy McKanna, Joachim Van Keirsbilck, Philippe Moerman, Andrea Poretti, Ratna Puri, Hilde Van Esch, Joseph G Gleeson, Enza Maria Valente

**Affiliations:** 1 Laboratory for Pediatric Brain Disease, New York Genome Center, Howard Hughes Medical Institute, The Rockefeller University, New York, New York, USA; 2 IRCCS Casa Sollievo della Sofferenza, Mendel Institute, San Giovanni Rotondo, Italy; 3 Department of Human Genetics, Laboratory for the Genetics of Cognition, Center for Human Genetics, KU Leuven, Belgium; 4 Department of Neurosciences, University of California San Diego (UCSD), La Jolla, California, USA; 5 Department of Biological and Environmental Science, University of Messina, Messina, Italy; 6 Department of Pediatrics, College of Medicine and Health Sciences, United Arab Emirates University, Al Ain, Abu Dhabi, United Arab Emirates; 7 Medical Genetics Department, İstanbul Medical Faculty, İstanbul University, İstanbul, Turkey; 8 Division of Pediatric Neurology, University Children's Hospital, Zurich, Switzerland; 9 Developmental Neurology Division, Fondazione IRCCS Istituto Neurologico Carlo Besta, Milan, Italy; 10 Department of Obstetrics and Gynecology, University Hospitals Leuven, Leuven, Belgium; 11 Department of Obstetrics and Gynecology, AZ Groeninge, Kortrijk, Belgium; 12 Medical Genetics Department, Koç University School of Medicine (KUSOM), Istanbul, Turkey; 13 Spectrum Health Medical Genetics, Grand Rapids, Michigan, USA; 14 Department of Child and Adolescent Neurology, National Institute Mongi Ben Hmida of Neurology of Tunis, La Rabta, Tunisia; 15 Department of Pathology, University Hospitals Leuven, Leuven, Belgium; 16 Section of Pediatric Neuroradiology, Division of Pediatric Radiology, The Russell H. Morgan Department of Radiology and Radiological Science, The Johns Hopkins School of Medicine, Baltimore, Maryland, USA; 17 Center of Medical Genetics, Sir Ganga Ram Hospital, Rajinder Nagar, New Delhi, India; 18 Neurogenetics Laboratory, Howard Hughes Medical Institute, Chevy Chase, Maryland, USA; 19 Section of Neurosciences, Department of Medicine and Surgery, University of Salerno, Salerno, Italy

**Keywords:** Clinical genetics, Developmental, Genetics, Molecular genetics, Neurosciences

## Abstract

**Background:**

Ciliopathies are an extensive group of autosomal recessive or X-linked disorders with considerable genetic and clinical overlap, which collectively share multiple organ involvement and may result in lethal or viable phenotypes. In large numbers of cases the genetic defect remains yet to be determined. The aim of this study is to describe the mutational frequency and phenotypic spectrum of the *CEP120* gene.

**Methods:**

Exome sequencing was performed in 145 patients with Joubert syndrome (JS), including 15 children with oral-facial-digital syndrome type VI (OFDVI) and 21 Meckel syndrome (MKS) fetuses. Moreover, exome sequencing was performed in one fetus with tectocerebellar dysraphia with occipital encephalocele (TCDOE), molar tooth sign and additional skeletal abnormalities. As a parallel study, 346 probands with a phenotype consistent with JS or related ciliopathies underwent next-generation sequencing-based targeted sequencing of 120 previously described and candidate ciliopathy genes.

**Results:**

We present six probands carrying nine distinct mutations (of which eight are novel) in the *CEP120* gene, previously found mutated only in Jeune asphyxiating thoracic dystrophy (JATD). The CEP120-associated phenotype ranges from mild classical JS in four patients to more severe conditions in two fetuses, with overlapping features of distinct ciliopathies that include TCDOE, MKS, JATD and OFD syndromes. No obvious correlation is evident between the type or location of identified mutations and the ciliopathy phenotype.

**Conclusion:**

Our findings broaden the spectrum of phenotypes caused by *CEP120* mutations that account for nearly 1% of patients with JS as well as for more complex ciliopathy phenotypes. The lack of clear genotype–phenotype correlation highlights the relevance of comprehensive genetic analyses in the diagnostics of ciliopathies.

## Introduction

Ciliopathies are an extensive group of autosomal recessive or X-linked disorders caused by defects of the primary cilium, a highly conserved subcellular organelle found in most vertebrate cell types. Pathogenic mutations in genes required for proper processes involved at the primary cilium lead to disorders among which are cystic kidneys, retinal degeneration, intellectual disability, infertility and skeletal alterations. There is considerable genetic and clinical overlap among distinct ciliopathy syndromes that collectively share multiple organ involvement and may result in lethal or viable phenotypes.^1^

One of the most frequent ciliopathies is Joubert syndrome ((JS) MIM213300) that is uniquely characterised by a malformation of the cerebellum and brainstem known as the ‘molar tooth sign’ (MTS). This feature is defined by an abnormally deep interpeduncular fossa, elongated, thick and maloriented superior cerebellar peduncles and absent or hypoplastic cerebellar vermis, together giving the appearance of a ‘molar tooth’ on axial brain MRI through the junction of the midbrain and hindbrain (isthmus region). Besides this pathognomonic feature, the clinical and neuroimaging phenotype of JS and related disorders is heterogeneous, with frequent involvement of retina, kidneys, liver and skeleton and variable occurrence of additional brain malformations such as polymicrogyria, corpus callosum dysgenesis, occipital encephalocele and tectal dysplasia.^2–4^ The association of tectal and cerebellar dysraphia with occipital encephalocele (TCDOE) was first described by Friede in 1978.^5^ Only a handful of cases with TCDOE have been reported so far in the literature and the underlying genetic mechanism remains undetermined. Based on conventional neuroimaging and diffusion tensor imaging findings, TCDOE was recently suggested to be part of the JS spectrum.^6^

The severe end of the range of ciliopathies is represented by Meckel syndrome ((MKS), MIM249000), a lethal disorder characterised by cystic dysplastic kidneys, bile duct proliferation of the liver, occipital encephalocele, postaxial polydactyly, pulmonary hypoplasia due to oligohydramnios and occasional skeletal involvement such as bowing of long bones.^7^ Of the 12 genes identified as causative of MKS, 9 are also associated with JS.^8^ In addition, several families with occurrence of JS and MKS in the same sibship have been reported,^9^ ^10^ which confirms that MKS and JS are allelic disorders.

Despite the impressive acceleration in gene discovery provided in recent years by the advent of exome sequencing, it is estimated that the currently known ciliopathy genes are responsible only for approximately 55%–60% of JS and up to 70% of patients with MKS,^2^ ^11^ while a genetic diagnosis is not reached in the remaining individuals.

Recently, mutations in *CEP120* were reported in four individuals diagnosed with Jeune asphyxiating thoracic dystrophy (JATD), a skeletal ciliopathy.^12^ Here we report the identification of mutations in the *CEP120* gene in a spectrum of phenotypes ranging from mild classical JS to more severe conditions with overlapping features of distinct ciliopathies that include MKS, JATD and oral-facial-digital (OFD) syndromes. In addition, we propose *CEP120* mutations as a genetic cause of TCDOE.

## Patients and methods

### Patients

Two large cohorts of patients with a diagnosis of JS or MKS were selected at The Rockefeller University (New York, USA) and the CSS-Mendel Institute (Rome, Italy). The unique inclusion criterion for JS was the presence of the MTS on neuroimaging; MKS was diagnosed in presence of at least two of the classical signs (occipital encephalocele, renal cystic dysplasia, postaxial polydactyly) and a normal karyotype. Detailed clinical data were obtained from the referring clinicians who filled in a standardised questionnaire. Neuroimaging studies were qualitatively reviewed for the presence of supratentorial and infratentorial morphological brain anomalies. All families provided written informed consent, according to institutional guidelines. Ethics approval has been obtained by the Ethics Committees of the Rockefeller University, the IRCCS Casa Sollievo della Sofferenza Institute and the UZ Leuven.

### Exome sequencing

Exome sequencing was performed in 145 patients with JS, including 15 children with OFD syndrome type VI (OFDVI), and in 21 MKS fetuses. Moreover, exome sequencing was performed in one fetus with TCDOE, the MTS and additional skeletal abnormalities.

In solution, exome capture was carried out using the SureSelect Human All Exome 50 Mb Kit (Agilent Technologies, Santa Clara, California, USA) with 150 bp paired-end read sequences generated on a HiSeq2000 (Illumina, San Diego, California, USA). Sequences were aligned to hg19 and variants identified through the Genome Analysis Toolkit (GATK) pipeline.^13^ Variations were annotated with *in-house* software and the SeattleSeq server.^14^ Identified variants were checked against public databases dnSNP146, Exome Variant Server and ExAC. Currently known JS, MKS and OFD genes were specifically analysed for the presence of pathogenic mutations.

### Target resequencing of 120 ciliary genes

In order to assess the mutational frequency and phenotypic spectrum of *CEP120* mutations, this gene was included in a panel of 120 previously described and candidate ciliopathy genes (among which all known JS, MKS and OFD genes) used for next-generation sequencing-based targeted sequencing in a cohort of 346 probands with a phenotype consistent with JS or related ciliopathies. Targeted resequencing was performed on a Solid 5500xL platform using the TargetSeq Custom Enrichment System (Thermo Fisher Scientific), according to manufacturers' protocols. Briefly, DNA libraries were fragmented and coupled with specific adaptors, enriched for the set of genes of interest, barcoded for pooling and then amplified by emulsion PCR, prior to high-throughput sequencing. Variants were detected by means of the HaplotypeCaller software package of the GATK suite and filtered so that to include only variants covered by at least 20 reads and with mapping quality values exceeding a Phred-score of 30. Variants reported as validated polymorphisms with minor allele frequency (MAF) of ≥0.01 in publicly available human variation resources (dbSNP146, 1000 Genomes, National Heart, Lung, and Blood Institute Exome Sequencing Project Exome Variant Server (EVS)) as well as variants present in in-house control individuals with MAF of ≥0.01 were filtered out.

### Validation of *CEP120* variants

All identified *CEP120* variants were verified by bidirectional Sanger sequencing in the proband as well as available family members, to check segregation with the disease and carrier status of the respective parents. Potential pathogenicity was predicted using the software tools SIFT, PolyPhen-2, PROVEAN and Mutation Assessor. Conservation of affected residues was assessed by the Clustal Omega software. Reference sequences applied were *CEP120* gene, NM_153223.3, CEP120 protein and NP_694955.2. All identified variants have been submitted to the Leiden Open (source) Variation Database (LOVD) gene variant database.

Websites for all software and databases are listed in online supplementary resources section.

10.1136/jmedgenet-2016-103832.supp1 Supplementary resources section

## Results

### Identification of *CEP120* mutations

**Figure 1:**
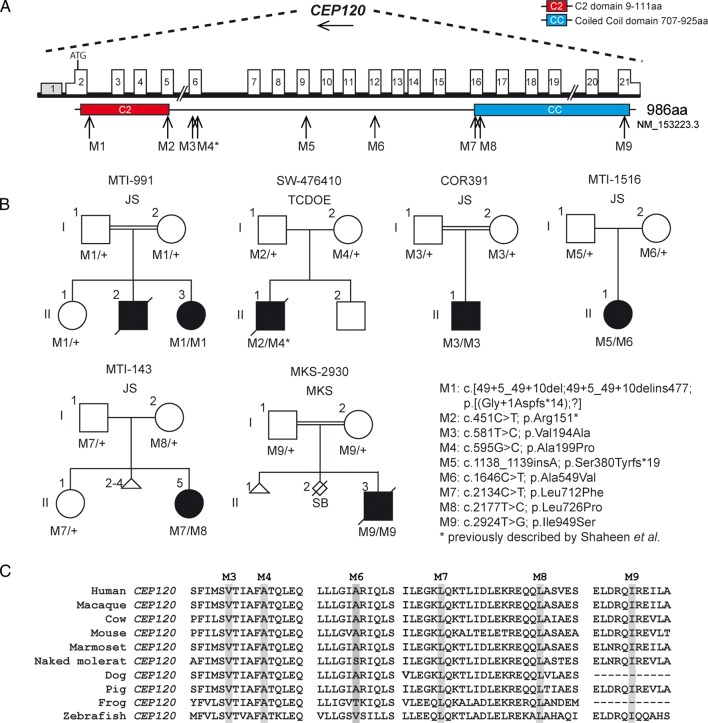
Pedigrees and schematic representation of *CEP120*. (A) Genomic structure and mRNA transcript of *CEP120* full-length isoform with 21 exons is shown. Untranslated regions (UTR's) are represented by half-height boxes. The location of the mutations is indicated by M1–M9. (B) Pedigrees of the affected families with ancestries of Italy (COR391), USA (MTI-143), Palestine (MTI-991), India (MTI-1516), Turkey (Meckel syndrome (MKS)-2930) and Belgium (SW-476410), respectively, is shown demonstrating the segregation of the compound heterozygous mutations in non-consanguineous families and homozygous mutations in consanguineous families. (C) Evolutionary conservation of affected amino acid residues in *CEP120* is shown. The mutated amino acids are indicated in grey and completely conserved in all species shown. JS, Joubert syndrome; M, mutation; TCDOE, tectocerebellar dysraphia with occipital encephalocele. See online supplementary figure S1 for the chromatograms of mutation M1 in *CEP120* (remaining mutation data not shown).

Biallelic mutations in the *CEP120* gene were identified in four probands with JS and two fetuses with overlapping ciliopathy phenotypes, all segregating with the disease within the families (figure 1A, B; three variants were present in homozygous and six in compound heterozygous state). Overall, nine distinct mutations were detected in this study, two of which were predicted to result in a truncated protein (one nonsense mutation and one 1 bp deletion). One of the variants comprised a splice-site mutation and the remaining six were missense mutations affecting highly conserved residues, all consistently predicted to be deleterious by four distinct *in silico* predictors (see figure 1C and online supplementary table S1). One of the missense mutations identified in this study (p.Ala199Pro) was previously reported by Shaheen and collaborators*.*^12^ Seven out of nine mutations were absent from the public reference databases (dbSNP, EVS and ExAC), while two of them (p.Ala199Pro and p.Leu712Phe) were found in dbSNP (rs114280473 and rs367600930, respectively), ExAC or EVS, however, at very low frequency (MAF<0.5%) and never in a homozygous state. All identified mutations were absent from our combined in-house database.

10.1136/jmedgenet-2016-103832.supp2 Supplementary table

10.1136/jmedgenet-2016-103832.supp3 Supplementary figure

Interestingly, one of the mutations consisted of a homozygous intronic deletion leading to intron retention in family MTI-991. Located at 5 bp from the splice donor site of exon 2, a 6 bp deletion led to the use of an alternative splice site 483 bp downstream (c.49+5_49+10delins477), in approximately 5% of mRNA, which was predicted to result in a truncated protein after 14 amino acids (p.Gly+1Aspfs*14) (see online supplementary figure S1). The remaining mRNA product showed normal splicing from exon 2 to exon 3. Mutations in the known genes causative of JS and other ciliopathies were excluded in all patients with *CEP120* mutations.

### Clinical features of mutated subjects

**Figure 2:**
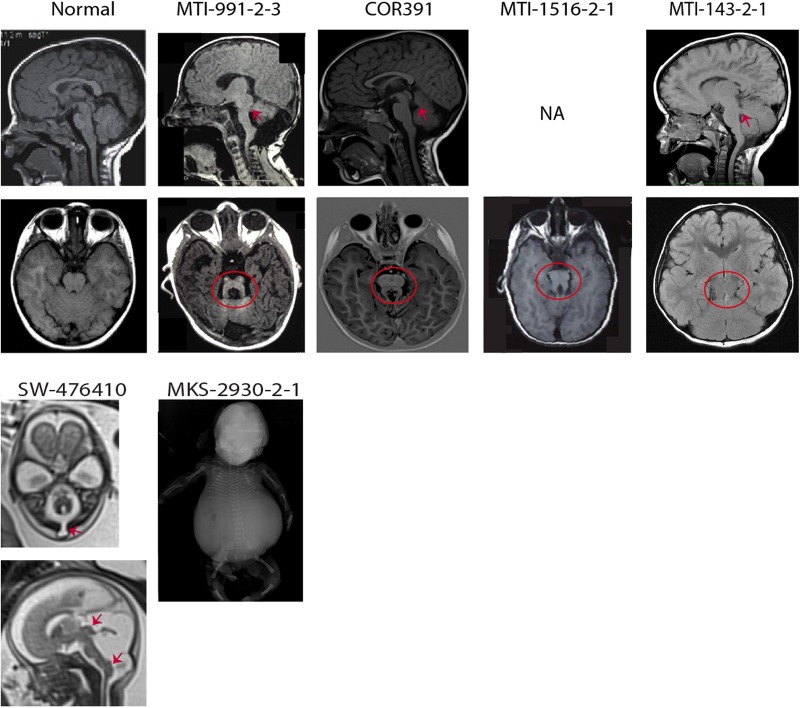
Neuroimaging or neuropathological findings of probands with *CEP120* mutations are shown. Upper panel: Sagittal T1-weighted and axial T1-weighted/coronal fluid attenuation inversion recovery MRIs of a healthy subject and patients with Joubert syndrome (JS) having *CEP120* mutations show thickened and maloriented superior cerebellar peduncle (upper arrows in MTI-991-2-3 and MTI-143-2-1), rostral shifting of the fastigium of the fourth ventricle (upper arrow in COR391), deepened interpeduncular fossa and constituting the ‘molar tooth sign’ (circled). Lower panel: SW-476410: axial and midsagittal T2-weighted fetal MRIs at 23 weeks of gestation show a suboccipital encephalocele (arrow on the axial image), severe hypoplasia of the cerebellar vermis and tectal dysplasia (upper arrow on the sagittal image) consistent with the diagnosis of tectocerebellar dysraphia with occipital encephalocele (TCDOE). In addition, enlargement of the posterior fossa and dorsal protuberance of the lower brainstem (lower arrow on the sagittal image) are noted; Meckel syndrome (MKS)-2930-2-1: a fetogram after termination of pregnancy in the second trimester reveals a marked abdominal distention, a narrow bell-shaped thorax with short ribs, rhizomelic limb shortening and bowing of long bones.

The clinical and neuroimaging features of the four probands with JS carrying *CEP120* mutations are summarised in table 1 and figure 2. They all presented with a neurological phenotype consisting of hypotonia, developmental delay and cognitive impairment. Ataxia and neonatal breathing abnormalities were reported in two individuals (MTI-143, MTI-1516), while abnormal ocular movements were observed in a single family. There was no involvement of other organs such as the retina, kidneys, liver and skeleton.

**Table 1:** Clinical, neuroimaging and genetic features of children with JS having *CEP120* mutations

Family	COR391	MTI-143	MTI-991	MTI-1516
Affected individuals (sex)	1 (F)	1 (M)	1 (M)	1 (F)
Age at last examination	4 years+7 months	11 years	2 years+1 month	2 years
Consanguinity	Yes	No	Yes	No
Country of origin	Italy	USA	Palestine	India
Nucleotide change	c.581T>C hom	c.2177T>C/c.2134C>T	c.(49+5_49+10del; 49+5_49+ 10delins477) hom	c.1138_1139insA/c.1646C>T
Protein change	p.Val194Ala hom	p. Leu726Pro/p.Leu712Phe	p.(Gly+1Aspfs*14;?)	p.Ser380Thrfs*19/p.Ala549Val
LOVD screening ID	00058830	00058831	00058832	00058833
LOVD individual ID	0000058794	0000058793	0000058796	0000058797
Neurological signs				
Hypotonia	Yes	Yes	Yes	Yes
Developmental delay/cognitive impairment	Yes	Yes	Yes	Yes
Abnormal breathing	No	Yes	No	Yes
Abnormal ocular movements	No	OMA, nystagmus, Duane syndrome	No	Strabismus
Truncal ataxia	Yes	No	Yes	No
Other organ involvement				
Retinal	No	No	No	No
Renal	No	No*	No	No
Hepatic	No	No	No	No
Oral-facial	No	No	No	No
Skeletal	No	No	No	No
Neuroimaging				
Molar tooth sign	Yes	Yes	Yes	Yes
Other	Mild ventriculomegaly	CC hypoplasia	No	No

*Grade II-III hydronephrosis was detected at birth but it spontaneously resolved after few months. No renal problems have been reported since then.

CC, corpus callosum; F, female; JS, Joubert syndrome; LOVD, Leiden Open (source) Variation Database; M, male; OMA, ocular motor apraxia.

Furthermore, mutations in *CEP120* were detected in two fetuses (table 2). The first one (MKS-2930) received the diagnosis of MKS in the second trimester of pregnancy, due to the presence of cystic dysplastic kidneys, occipital encephalocele and polydactyly. In addition, postmortem examination after pregnancy termination disclosed a narrow bell-shaped thorax with short ribs, rhizomelic limb shortening, bowing of long bones, microphthalmia, oral-facial defects (lobulated tongue with multiple frenula and cleft soft palate), ambiguous genitalia and anal atresia. Data on liver involvement were not available.

**Table 2:** Pathological and genetic features of fetuses with *CEP120* mutations

Family	MKS-2930	SW-476410
Affected individuals (sex)	1 (M)*	1(M)
Pregnancy termination	23 gw	27 gw
Consanguinity	Yes	No
Country of origin	Turkey	Belgium
Nucleotide change	c.2924T>G hom	c.451C>T/c.595G>C
Protein change	p.Ile975Ser hom	p.Arg151*/ p.Ala199Pro
LOVD screening ID	00058834	00058835
LOVD individual ID	0000058798	0000058799
Central nervous system		
Molar tooth sign	n.a.	Yes
Enlarged posterior fossa	Yes	Yes
Occipital/suboccipital encephalocele	Yes	Yes
Dysplastic tectum	n.a.	Yes
Skeletal features		
Bell-shaped thorax with short ribs	Yes	Yes
Rhizomelic limb shortening	Yes	Yes (mild)
Polydactyly	Post-ax LH, pre-ax feet	No
Other digital anomalies	Brachydactyly, clinodactyly, sandal gap	No
Bowing of long bones	Yes	No
Other organ involvement		
Eyes	Microphthalmia	No
Kidneys	Cystic kidneys	No
Liver	n.a.	No
Oral-facial	Lobulated tongue, cleft palate, multiple lingual frenula	Cleft palate
Other	Ambiguous genitalia, anal atresia	No

*This family previously had one spontaneous abortion at 6 gw and a stillborn baby showing encephalocele, microphthalmia, rhizomelic limb shortening and polydactyly. No autopsy was performed.

ax, axial; gw, gestational weeks; LH, left hand; LOVD, Leiden Open (source) Variation Database; MKS, Meckel syndrome; n.a., not available.

The second fetus (SW-476410) was diagnosed in utero with TCDOE in the spectrum of JS, due to the presence of a suboccipital encephalocele, dysplastic tectum, severe hypoplasia of the cerebellar vermis and the MTS, as shown by fetal MRI at 23 weeks of gestation. In addition, postmortem examination revealed cleft palate, narrow thorax with short ribs and secondary lung hypoplasia and discrete rhizomelic shortening of limbs. Liver and kidneys were normal and no polydactyly was observed.

## Discussion

We report for the first time the occurrence of *CEP120* mutations in 4 out of 491 patients with JS (0.8%). Of note, all of them presented with a mild, purely neurological phenotype, adding *CEP120* to the growing list of ciliary genes that are associated with this relatively benign phenotype.^3^

Furthermore, we identified *CEP120* mutations in two fetuses (MKS-2930 and SW-476410) with more complex phenotypes and overlapping features of distinct ciliopathies. Both showed central nervous system malformations characterised by occipital/suboccipital encephalocele and enlarged posterior fossa. In addition, one fetus (SW-476410) showed marked hypoplasia of the cerebellar vermis, dysplasia of the tectal plate and a posterior protuberance of the lower brainstem.

The association of hypoplasia or agenesis of the cerebellar vermis, tectal malformation (reminiscent of the tectal beaking characteristic of the Chiari type 2 malformation) and occipital encephalocele was first described by Padget and Lindburg in 1972.^15^ In 1978, Friede introduced the term ‘tectocerebellar dysraphia with occipital encephalocele’ (TCDOE) to describe this constellation of findings.^5^ To our knowledge, in literature this entity has only been described as a clinical diagnosis in eight cases.^6^ ^16–21^ Neuroimaging studies showed elongated and horizontally orientated superior cerebellar peduncles reminiscent of the MTS in all reported patients and a deepened interpeduncular fossa in three of them. In addition, diffusion tensor imaging in one child revealed absence of the decussation of the cerebellar peduncles, as previously shown in patients with JS.^4–6^ In the fetus SW-476410, the suboccipital encephalocele was located at the level of the foramen magnum. This is an unusual location for encephalocele and has previously been reported only in JS.^4^ ^22^ Furthermore, in another published case, TCDOE was associated with a congenital heart disease and situs inversus, reinforcing the hypothesis of an underlying ciliopathy.^21^ However, no causative genes for the TCDOE phenotype had been found to date and, to our knowledge, this is the first report of mutations in a ciliary gene being causative of this exceptional condition. The finding of mutations in *CEP120* in one fetus with TCDOE further supports the observation that TCDOE represents a morphological phenotype within the spectrum of JS.

Interestingly, both *CEP120*-mutated fetuses showed a small, bell-shaped thorax with short ribs and rhizomelic shortening of limbs, which are typical features of the skeletal ciliopathy known as JATD. Besides these malformations, the typical JATD phenotype is characterised by polydactyly, cystic dysplastic kidneys and bile duct proliferation of the liver, all features that are also found within the spectrum of MKS.^23^ However, only one of the fetuses presented with postaxial and preaxial polydactyly and cystic kidneys and therefore was initially diagnosed with MKS.

The co-occurrence of JS and the core signs of JATD (mainly a small thorax with secondary respiratory distress), was previously described in some living patients.^23^ In addition, mutations in two genes, *CSPP1* and *KIAA0586*, were reported to cause both JS and JATD, either isolated or in variable combination.^24–31^ Recently, a founder missense mutation of *CEP120* (p.Ala199Pro) was identified in four Saudi Arabian families presenting the full-blown JATD phenotype, associated with cerebellar vermis hypoplasia in two patients and MTS in one of them.^12^ Remarkably, despite his Flemish origin without Arabian ancestors, one fetus in the present study (SW-476410) was found to carry the same founder mutation in combination with a nonsense mutation, leading to the hypothesis that this particular mutation might predispose to the development of skeletal defects.

Noteworthy, the two fetuses with JATD features in our study also showed oral-facial defects including cleft lip, tongue hamartomas and bifid or lobulated tongue. These oral-facial findings were also reported in three of four *CEP120*-mutated patients with JATD reported previously, suggesting that they may be commonly associated with the skeletal phenotype and specific to *CEP120* mutations in both lethal or surviving cases. The co-occurrence of features, each of which typical of distinct ciliopathies (short rib polydactyly, JS/MKS spectrum and OFD syndromes) in the same patient is an intriguing finding that has already been reported in the literature. For instance, Thomas and collaborators identified mutations in the *TCTN3* gene in individuals with classical JS and in fetuses with Mohr–Majewski syndrome (also called OFD IV): an extremely severe condition characterised by oral-facial features, polydactyly, encephalocele and MTS, as well as renal, hepatic and skeletal anomalies.^32^ Moreover, patients with JS can also present oral-facial defects and polydactyly (mainly preaxial and mesoaxial), a rare association termed OFDVI.^33^ So far, mutations in *TMEM216*, *OFD1* and *C5Orf42* were found mutated in a minority of patients with OFDVI.^9^ ^34–37^ In the present study, *CEP120* was sequenced in a cohort of 15 patients with OFDVI negative for mutations in currently known JS genes, but no pathogenic mutations were identified. This result suggests that *CEP120* may have little contribution to this specific JS phenotype.

Cep120 is a centrosomal protein that is essential for microtubule organisation and centriolar assembly and elongation.^38^ ^39^ In mouse fetal brain, Cep120 is expressed in neural progenitors during neocortical development and plays an important role in neuronal migration and maintenance of the neural progenitor pool.^40^ Its downregulation alters microtubule organisation and centrosome motility, with consequent defective axonal growth and impaired neuronal migration.^41^ Accordingly, the conditional ablation of Cep120 in the mouse central nervous system led to aberrant centriolar duplication, failed ciliogenesis and altered Sonic Hedgehog pathway activity in the granule neuron progenitors, resulting in hydrocephalus and severe cerebellar hypoplasia.^42^ In addition, splice-blocking morpholino injected zebrafish knocking down *Cep120* presented with a range of phenotypes including a centrally curved tail, hydrocephalus, otolith defects and craniofacial defects.^12^

Of note, Cep120 was found to interact with Talpid3,^42^ the homologue of human *KIAA0586*, in which mutations give rise to a similar spectrum of ciliopathies, ranging from a mild, pure JS to a more complex phenotype with overlapping features of JS and JATD.^27–31^ This increasing range of phenotypes associated with mutations in one and the same gene argues for the need of broad next-generation sequencing-based approaches, to allow the detection of novel, rare phenotypes associated with ciliary gene mutations.

The mechanism through which mutations in the same gene may cause such wide phenotypic variability remains unexplained. Genotype–phenotype correlations have been proposed for ciliopathy genes such as *TMEM67*, in which two loss-of-function mutations were found to cause MKS while the presence of at least one hypomorphic mutation resulted in non-lethal phenotypes.^43^ However, this is not obvious for *CEP120*, as the MKS fetus (MKS-2930) was homozygous for a missense mutation, while at least one JS patient (MTI-991) carried a splice-site mutation that was shown to introduce a premature stop codon in part of the product. Growing evidence is demonstrating that ciliopathies might undergo an oligogenic mode of inheritance and that heterozygous mutations in distinct genes can modulate the clinical expression of the main recessive mutations. For instance, the common variant p.Arg830Trp in the *AHI1* gene was found to significantly enhance the frequency of retinal disease in patients with *NPHP1*-associated nephronophthisis.^44^ The identification of such genetic modifiers, as well as of other factors able to influence the penetrance and expression of ciliary gene mutations, represents one of the biggest challenges of current research on ciliopathies, with significant implications for diagnosis, management and counselling of patients and their families.

In conclusion, this study adds *CEP120* to the growing list of causative genes for a variety of clinically distinct but overlapping ciliopathies. In addition, we provide the primary molecular diagnosis in a case of TCDOE and confirm the classification of this morphological phenotype within the spectrum of JS.

## References

[1] Hildebrandt F, Benzing T, Katsanis N Ciliopathies. N Engl J Med 2011;364:1533–43. 10.1056/NEJMra1010172 21506742 PMC3640822

[2] Bachmann-Gagescu R, Dempsey JC, Phelps IG, O'Roak BJ, Knutzen DM, Rue TC, Ishak GE, Isabella CR, Gorden N, Adkins J, Boyle EA, de Lacy N, O'Day D, Alswaid A, Ramadevi AR, Lingappa L, Lourenco C, Martorell L, Garcia-Cazorla A, Ozyurek H, Haliloglu G, Tuysuz B, Topcu M, University of Washington Center for Mendelian G, Chance P, Parisi MA, Glass IA, Shendure J, Doherty D Joubert syndrome: a model for untangling recessive disorders with extreme genetic heterogeneity. J Med Genet 2015;52:514–22. 10.1136/jmedgenet-2015-103087 26092869 PMC5082428

[3] Romani M, Micalizzi A, Valente EM Joubert syndrome: congenital cerebellar ataxia with the molar tooth. Lancet Neurol 2013;12:894–905. 10.1016/S1474-4422(13)70136-4 23870701 PMC3809058

[4] Poretti A, Huisman TA, Scheer I, Boltshauser E Joubert syndrome and related disorders: spectrum of neuroimaging findings in 75 patients. AJNR Am J Neuroradiol 2011;32:1459–63. 10.3174/ajnr.A2517 21680654 PMC7964342

[5] Friede RL, Boltshauser E Uncommon syndromes of cerebellar vermis aplasia. I: joubert syndrome. Dev Med Child Neurol 1978;20:758–63. 10.1111/j.1469-8749.1978.tb15307.x 729929

[6] Poretti A, Singhi S, Huisman TA, Meoded A, Jallo G, Ozturk A, Boltshauser E, Tekes A Tecto-cerebellar dysraphism with occipital encephalocele: not a distinct disorder, but part of the Joubert syndrome spectrum? Neuropediatrics 2011;42:170–4. 10.1055/s-0031-1287763 21932183

[7] Salonen R The Meckel syndrome: clinicopathological findings in 67 patients. Am J Med Genet 1984;18:671–89. 10.1002/ajmg.1320180414 6486167

[8] Barker AR, Thomas R, Dawe HR Meckel-Gruber syndrome and the role of primary cilia in kidney, skeleton, and central nervous system development. Organogenesis 2014;10:96–107. 10.4161/org.27375 24322779 PMC4049900

[9] Valente EM, Logan CV, Mougou-Zerelli S, Lee JH, Silhavy JL, Brancati F, Iannicelli M, Travaglini L, Romani S, Illi B, Adams M, Szymanska K, Mazzotta A, Lee JE, Tolentino JC, Swistun D, Salpietro CD, Fede C, Gabriel S, Russ C, Cibulskis K, Sougnez C, Hildebrandt F, Otto EA, Held S, Diplas BH, Davis EE, Mikula M, Strom CM, Ben-Zeev B, Lev D, Sagie TL, Michelson M, Yaron Y, Krause A, Boltshauser E, Elkhartoufi N, Roume J, Shalev S, Munnich A, Saunier S, Inglehearn C, Saad A, Alkindy A, Thomas S, Vekemans M, Dallapiccola B, Katsanis N, Johnson CA, Attie-Bitach T, Gleeson JG Mutations in TMEM216 perturb ciliogenesis and cause Joubert, Meckel and related syndromes. Nat Genet 2010;42:619–25. 10.1038/ng.594 20512146 PMC2894012

[10] Brancati F, Iannicelli M, Travaglini L, Mazzotta A, Bertini E, Boltshauser E, D'Arrigo S, Emma F, Fazzi E, Gallizzi R, Gentile M, Loncarevic D, Mejaski-Bosnjak V, Pantaleoni C, Rigoli L, Salpietro CD, Signorini S, Stringini GR, Verloes A, Zabloka D, Dallapiccola B, Gleeson JG, Valente EM MKS3/TMEM67 mutations are a major cause of COACH Syndrome, a Joubert Syndrome related disorder with liver involvement. Hum Mutat 2009;30:E432–42. 10.1002/humu.20924 19058225 PMC2635428

[11] Shaheen R, Faqeih E, Alshammari MJ, Swaid A, Al-Gazali L, Mardawi E, Ansari S, Sogaty S, Seidahmed MZ, Almotairi MI, Farra C, Kurdi W, Al-Rasheed S, Alkuraya FS Genomic analysis of Meckel-Gruber syndrome in Arabs reveals marked genetic heterogeneity and novel candidate genes. Eur J Hum Genet 2013;21:762–8. 10.1038/ejhg.2012.254 23169490 PMC3722952

[12] Shaheen R, Schmidts M, Faqeih E, Hashem A, Lausch E, Holder I, Superti-Furga A, Consortium UK, Mitchison HM, Almoisheer A, Alamro R, Alshiddi T, Alzahrani F, Beales PL, Alkuraya FS A founder CEP120 mutation in Jeune asphyxiating thoracic dystrophy expands the role of centriolar proteins in skeletal ciliopathies. Hum Mol Genet 2015;24:1410–19. 10.1093/hmg/ddu555 25361962 PMC4321448

[13] DePristo MA, Banks E, Poplin R, Garimella KV, Maguire JR, Hartl C, Philippakis AA, del Angel G, Rivas MA, Hanna M, McKenna A, Fennell TJ, Kernytsky AM, Sivachenko AY, Cibulskis K, Gabriel SB, Altshuler D, Daly MJ A framework for variation discovery and genotyping using next-generation DNA sequencing data. Nat Genet 2011;43:491–8. 10.1038/ng.806 21478889 PMC3083463

[14] Dixon-Salazar T, Silhavy JL, Marsh SE, Louie CM, Scott LC, Gururaj A, Al-Gazali L, Al-Tawari AA, Kayserili H, Sztriha L, Gleeson JG Mutations in the AHI1 gene, encoding jouberin, cause Joubert syndrome with cortical polymicrogyria. Am J Hum Genet 2004;75:979–87. 10.1086/425985 15467982 PMC1182159

[15] Padget DH, Lindenberg R Inverse cerebellum morphogenetically related to Dandy-Walker and Arnold-Chiari syndromes: bizarre malformed brain with occipital encephalocele. Johns Hopkins Med J 1972;131:228–46. 5075526

[16] Anik I, Koc K, Anik Y, Yildiz DK, Ceylan S Tectocerebellar dysraphism with vermian encephalocele. J Child Neurol 2010;25:1411–14. 10.1177/0883073810367828 20445194

[17] Dehdashti AR, Abouzeid H, Momjian S, Delavelle J, Rilliet B Occipital extra- and intracranial lipoencephalocele associated with tectocerebellar dysraphia. Childs Nerv Syst 2004;20:225–8; discussion 29–38 10.1007/s00381-003-0867-1 14986040

[18] Demaerel P, Kendall BE, Wilms G, Halpin SF, Casaer P, Baert AL Uncommon posterior cranial fossa anomalies: MRI with clinical correlation. Neuroradiology 1995;37:72–6. 10.1007/BF00588525 7708195

[19] Jaspan T New concepts on posterior fossa malformations. Pediatr Radiol 2008;38(Suppl 3):S409–14. 10.1007/s00247-008-0848-3 18470450

[20] Komiyama A, Toda H, Johkura K, Kataoka M, Yamamoto I Pretectal pseudobobbing associated with an expanding posterior fossa cyst in tectocerebellar dysraphia: an electro-oculographic study. J Neurol 1999;246:221–3. 10.1007/s004150050338 10323322

[21] Krishnamurthy S, Kapoor S, Sharma V, Prakash A Tectocerebellar dysraphia and occipital encephalocele: an unusual association with abdominal situs inversus and congenital heart disease. Indian J Pediatr 2008;75:1178–80. 10.1007/s12098-008-0183-6 18810345

[22] Doherty D Joubert syndrome: insights into brain development, cilium biology, and complex disease. Semin Pediatr Neurol 2009;16:143–54. 10.1016/j.spen.2009.06.002 19778711 PMC2804071

[23] Lehman AM, Eydoux P, Doherty D, Glass IA, Chitayat D, Chung BY, Langlois S, Yong SL, Lowry RB, Hildebrandt F, Trnka P Co-occurrence of Joubert syndrome and Jeune asphyxiating thoracic dystrophy. Am J Med Genet A 2010;152A:1411–19. 10.1002/ajmg.a.33416 20503315 PMC4048012

[24] Tuz K, Bachmann-Gagescu R, O'Day DR, Hua K, Isabella CR, Phelps IG, Stolarski AE, O'Roak BJ, Dempsey JC, Lourenco C, Alswaid A, Bonnemann CG, Medne L, Nampoothiri S, Stark Z, Leventer RJ, Topcu M, Cansu A, Jagadeesh S, Done S, Ishak GE, Glass IA, Shendure J, Neuhauss SC, Haldeman-Englert CR, Doherty D, Ferland RJ Mutations in CSPP1 cause primary cilia abnormalities and Joubert syndrome with or without Jeune asphyxiating thoracic dystrophy. Am J Hum Genet 2014;94:62–72. 10.1016/j.ajhg.2013.11.019 24360808 PMC3882733

[25] Akizu N, Silhavy JL, Rosti RO, Scott E, Fenstermaker AG, Schroth J, Zaki MS, Sanchez H, Gupta N, Kabra M, Kara M, Ben-Omran T, Rosti B, Guemez-Gamboa A, Spencer E, Pan R, Cai N, Abdellateef M, Gabriel S, Halbritter J, Hildebrandt F, van Bokhoven H, Gunel M, Gleeson JG Mutations in CSPP1 lead to classical Joubert syndrome. Am J Hum Genet 2014;94:80–6. 10.1016/j.ajhg.2013.11.015 24360807 PMC3882909

[26] Shaheen R, Shamseldin HE, Loucks CM, Seidahmed MZ, Ansari S, Ibrahim Khalil M, Al-Yacoub N, Davis EE, Mola NA, Szymanska K, Herridge W, Chudley AE, Chodirker BN, Schwartzentruber J, Majewski J, Katsanis N, Poizat C, Johnson CA, Parboosingh J, Boycott KM, Innes AM, Alkuraya FS Mutations in CSPP1, encoding a core centrosomal protein, cause a range of ciliopathy phenotypes in humans. Am J Hum Genet 2014;94:73–9. 10.1016/j.ajhg.2013.11.010 24360803 PMC3882732

[27] Bachmann-Gagescu R, Phelps IG, Dempsey JC, Sharma VA, Ishak GE, Boyle EA, Wilson M, Marques Lourenco C, Arslan M, University of Washington Center for Mendelian Genomics, Shendure J, Doherty D KIAA0586 is Mutated in Joubert Syndrome. Hum Mutat 2015;36:831–5. 10.1002/humu.22821 26096313 PMC4537327

[28] Stephen LA, Tawamie H, Davis GM, Tebbe L, Nurnberg P, Nurnberg G, Thiele H, Thoenes M, Boltshauser E, Uebe S, Rompel O, Reis A, Ekici AB, McTeir L, Fraser AM, Hall EA, Mill P, Daudet N, Cross C, Wolfrum U, Jamra RA, Davey MG, Bolz HJ TALPID3 controls centrosome and cell polarity and the human ortholog KIAA0586 is mutated in Joubert syndrome (JBTS23). Elife 2015;4:pii: e08077 10.7554/eLife.08077 PMC4641851 26386247

[29] Malicdan MC, Vilboux T, Stephen J, Maglic D, Mian L, Konzman D, Guo J, Yildirimli D, Bryant J, Fischer R, Zein WM, Snow J, Vemulapalli M, Mullikin JC, Toro C, Solomon BD, Niederhuber JE, Program NCS, Gahl WA, Gunay-Aygun M Mutations in human homologue of chicken talpid3 gene (KIAA0586) cause a hybrid ciliopathy with overlapping features of Jeune and Joubert syndromes. J Med Genet 2015;52:830–9. 10.1136/jmedgenet-2015-103316 26386044 PMC5517294

[30] Alby C, Piquand K, Huber C, Megarbane A, Ichkou A, Legendre M, Pelluard F, Encha-Ravazi F, Abi-Tayeh G, Bessieres B, El Chehadeh-Djebbar S, Laurent N, Faivre L, Sztriha L, Zombor M, Szabo H, Failler M, Garfa-Traore M, Bole C, Nitschke P, Nizon M, Elkhartoufi N, Clerget-Darpoux F, Munnich A, Lyonnet S, Vekemans M, Saunier S, Cormier-Daire V, Attie-Bitach T, Thomas S Mutations in KIAA0586 cause lethal ciliopathies ranging from a hydrolethalus phenotype to short-rib polydactyly syndrome. Am J Hum Genet 2015;97:311–18. 10.1016/j.ajhg.2015.06.003 26166481 PMC4573270

[31] Roosing S, Hofree M, Kim S, Scott E, Copeland B, Romani M, Silhavy JL, Rosti RO, Schroth J, Mazza T, Miccinilli E, Zaki MS, Swoboda KJ, Milisa-Drautz J, Dobyns WB, Mikati MA, Incecik F, Azam M, Borgatti R, Romaniello R, Boustany RM, Clericuzio CL, D'Arrigo S, Stromme P, Boltshauser E, Stanzial F, Mirabelli-Badenier M, Moroni I, Bertini E, Emma F, Steinlin M, Hildebrandt F, Johnson CA, Freilinger M, Vaux KK, Gabriel SB, Aza-Blanc P, Heynen-Genel S, Ideker T, Dynlacht BD, Lee JE, Valente EM, Kim J, Gleeson JG Functional genome-wide siRNA screen identifies KIAA0586 as mutated in Joubert syndrome. Elife 2015;4:e06602 10.7554/eLife.06602 26026149 PMC4477441

[32] Thomas S, Legendre M, Saunier S, Bessieres B, Alby C, Bonniere M, Toutain A, Loeuillet L, Szymanska K, Jossic F, Gaillard D, Yacoubi MT, Mougou-Zerelli S, David A, Barthez MA, Ville Y, Bole-Feysot C, Nitschke P, Lyonnet S, Munnich A, Johnson CA, Encha-Razavi F, Cormier-Daire V, Thauvin-Robinet C, Vekemans M, Attie-Bitach T TCTN3 mutations cause Mohr-Majewski syndrome. Am J Hum Genet 2012;91:372–8. 10.1016/j.ajhg.2012.06.017 22883145 PMC3415538

[33] Poretti A, Vitiello G, Hennekam RC, Arrigoni F, Bertini E, Borgatti R, Brancati F, D'Arrigo S, Faravelli F, Giordano L, Huisman TA, Iannicelli M, Kluger G, Kyllerman M, Landgren M, Lees MM, Pinelli L, Romaniello R, Scheer I, Schwarz CE, Spiegel R, Tibussek D, Valente EM, Boltshauser E Delineation and diagnostic criteria of Oral-Facial-Digital Syndrome type VI. Orphanet J Rare Dis 2012;7:4 10.1186/1750-1172-7-4 22236771 PMC3313869

[34] Lopez E, Thauvin-Robinet C, Reversade B, Khartoufi NE, Devisme L, Holder M, Ansart-Franquet H, Avila M, Lacombe D, Kleinfinger P, Kaori I, Takanashi JI, Le Merrer M, Martinovic J, Noel C, Shboul M, Ho L, Guven Y, Razavi F, Burglen L, Gigot N, Darmency-Stamboul V, Thevenon J, Aral B, Kayserili H, Huet F, Lyonnet S, Le Caignec C, Franco B, Riviere JB, Faivre L, Attie-Bitach T C5orf42 is the major gene responsible for OFD syndrome type VI. Hum Genet 2014;133:367–77. 10.1007/s00439-013-1385-1 24178751

[35] Romani M, Mancini F, Micalizzi A, Poretti A, Miccinilli E, Accorsi P, Avola E, Bertini E, Borgatti R, Romaniello R, Ceylaner S, Coppola G, D'Arrigo S, Giordano L, Janecke AR, Lituania M, Ludwig K, Martorell L, Mazza T, Odent S, Pinelli L, Poo P, Santucci M, Signorini S, Simonati A, Spiegel R, Stanzial F, Steinlin M, Tabarki B, Wolf NI, Zibordi F, Boltshauser E, Valente EM Oral-facial-digital syndrome type VI: is C5orf42 really the major gene? Hum Genet 2015;134:123–6. 10.1007/s00439-014-1508-3 25407461 PMC4282684

[36] Thauvin-Robinet C, Cossée M, Cormier-Daire V, Van Maldergem L, Toutain A, Alembik Y, Bieth E, Layet V, Parent P, David A, Goldenberg A, Mortier G, Héron D, Sagot P, Bouvier AM, Huet F, Cusin V, Donzel A, Devys D, Teyssier JR, Faivre L Clinical, molecular, and genotype-phenotype correlation studies from 25 cases of oral-facial-digital syndrome type 1: a French and Belgian collaborative study. J Med Genet 2006;43:54–61. 10.1136/jmg.2004.027672 16397067 PMC2564504

[37] Del Giudice E, Macca M, Imperati F, D'Amico A, Parent P, Pasquier L, Layet V, Lyonnet S, Stamboul-Darmency V, Thauvin-Robinet C, Franco B, Oral-Facial-Digital Type ICG. CNS involvement in OFD1 syndrome: a clinical, molecular, and neuroimaging study. Orphanet J Rare Dis 2014;9:74 10.1186/1750-1172-9-74 24884629 PMC4113190

[38] Lin YN, Wu CT, Lin YC, Hsu WB, Tang CJ, Chang CW, Tang TK CEP120 interacts with CPAP and positively regulates centriole elongation. J Cell Biol 2013;202:211–19. 10.1083/jcb.201212060 23857771 PMC3718976

[39] Mahjoub MR, Xie Z, Stearns T Cep120 is asymmetrically localized to the daughter centriole and is essential for centriole assembly. J Cell Biol 2010;191:331–46. 10.1083/jcb.201003009 20956381 PMC2958470

[40] Xie Z, Moy LY, Sanada K, Zhou Y, Buchman JJ, Tsai LH Cep120 and TACCs control interkinetic nuclear migration and the neural progenitor pool. Neuron 2007;56:79–93. 10.1016/j.neuron.2007.08.026 17920017 PMC2642594

[41] de Anda FC, Meletis K, Ge X, Rei D, Tsai LH Centrosome motility is essential for initial axon formation in the neocortex. J Neurosci 2010;30: 10391–406. 10.1523/JNEUROSCI.0381-10.2010 20685982 PMC6634663

[42] Wu C, Yang M, Li J, Wang C, Cao T, Tao K, Wang B Talpid3-binding centrosomal protein Cep120 is required for centriole duplication and proliferation of cerebellar granule neuron progenitors. PLoS ONE 2014;9:e107943 10.1371/journal.pone.0107943 25251415 PMC4176001

[43] Iannicelli M, Brancati F, Mougou-Zerelli S, Mazzotta A, Thomas S, Elkhartoufi N, Travaglini L, Gomes C, Ardissino GL, Bertini E, Boltshauser E, Castorina P, D'Arrigo S, Fischetto R, Leroy B, Loget P, Bonniere M, Starck L, Tantau J, Gentilin B, Majore S, Swistun D, Flori E, Lalatta F, Pantaleoni C, Penzien J, Grammatico P, Dallapiccola B, Gleeson JG, Attie-Bitach T, Valente EM Novel TMEM67 mutations and genotype-phenotype correlates in meckelin-related ciliopathies. Hum Mutat 2010;31:E1319–31. 10.1002/humu.21239 20232449 PMC2918781

[44] Louie CM, Caridi G, Lopes VS, Brancati F, Kispert A, Lancaster MA, Schlossman AM, Otto EA, Leitges M, Grone HJ, Lopez I, Gudiseva HV, O'Toole JF, Vallespin E, Ayyagari R, Ayuso C, Cremers FP, den Hollander AI, Koenekoop RK, Dallapiccola B, Ghiggeri GM, Hildebrandt F, Valente EM, Williams DS, Gleeson JG AHI1 is required for photoreceptor outer segment development and is a modifier for retinal degeneration in nephronophthisis. Nat Genet 2010;42:175–80. 10.1038/ng.519 20081859 PMC2884967

