# Supplementary material for: Mutations in CEP120 cause Joubert syndrome as well as complex ciliopathy phenotypes
Source: J Med Genet. 2016 May 6;53(9):608–15. doi: 10.1136/jmedgenet-2016-103832 (PMC5013089; doi:10.1136/jmedgenet-2016-103832)
Supplement: Supplementary resources section [file jmedgenet-2016-103832supp_Online-Supplementary-resources.pdf]

Supplemental material

Online resources

dbSNP: http://www.ncbi.nlm.nih.gov/projects/SNP; 1000 Genomes: http://browser.1000genomes.org; Exome Aggregation Consortium [ExAC]: http://exac.broadinstitute.org/; NHLBI Exome Sequencing Project Exome Variant Server [EVS]: http://evs.gs.washington.edu/EVS/; PolyPhen-2: http://genetics.bwh.harvard.edu/pph2/; SIFT: http://sift.jcvi.org/; Mutation Assessor: http://mutationassessor.org/; Provean: http://provean.jcvi.org; HaplotypeCaller and GATK: https://www.broadinstitute.org/gatk/; Clustal Omega: http://www.ebi.ac.uk/Tools/msa/clustalo/; LOVD: http://LOVD.nl/CEP120.
