# Supplementary material for: Mutations in CEP120 cause Joubert syndrome as well as complex ciliopathy phenotypes
Source: J Med Genet. 2016 May 6;53(9):608–15. doi: 10.1136/jmedgenet-2016-103832 (PMC5013089; doi:10.1136/jmedgenet-2016-103832)
Supplement: Supplementary table [file jmedgenet-2016-103832supp_table.pdf]

Supplemental Table 1 – Predicted pathogenicity of identified *CEP120* variants

	SIFT	Polyphen-2	Provean	MutationAssessor	dbSNP	EVS	ExAC
**p.Val194Ala**	0	0,999	Deleterious (-2.98)	M (2.32)	no	no	no
**p.Ala199Pro**	0	1	Deleterious (-3.40)	M (2.36)	rs367600930	0.024/0.0/0.0162 (no hom)	0.0041% (no hom)
**p.Leu712Phe**	0,1*	0,997	Deleterious (-3.37)	M (2.48)	rs114280473 (MAF 0.0018)	0.4302/0.1362/0.3306 (no hom)	0.003547 (no hom)
**p.Leu726Pro**	0	0,999	Deleterious (-5.11)	M (2.46)	no	No	no
**p.Ile975Ser**	0	0,998	Deleterious (-4.28)	L (1.89)	no	No	no

Legend: MAF: minor allele frequency; hom: homozygotes. Range and pathogenicity scores for each software are as follows: SIFT: range 0-1 (deleterious ≤0.05; tolerated >0.05); PolyPhen-2: range 0-1 (probably damaging ≥0.957; possibly damaging 0.453≥score≤0.957; benign ≤0.453); Provean: range -13 - +4 (deleterious≤-2.5; neutral>-2.5); Mutation Assessor: range -5.76 - +5.37 (neutral <0.6; low impact 0.6≥score<2.0, medium impact 2.0≥score<3.7, high impact ≥3.7).
