# Supplementary material for: Mutations in CEP120 cause Joubert syndrome as well as complex ciliopathy phenotypes
Source: J Med Genet. 2016 May 6;53(9):608–15. doi: 10.1136/jmedgenet-2016-103832 (PMC5013089; doi:10.1136/jmedgenet-2016-103832)
Supplement: Supplementary figure [file jmedgenet-2016-103832supp_figure.pdf]

Legend to Supplemental Figure 1

A) *CEP120* cDNA analysis of the effect of the M1 mutation on splicing of exon 5 in the patient cell lines versus control cell lines. Agarose gel electrophoresis revealed a 137bp product representing the normal transcript in all tested samples (forward primer *attgctcatcgtcgtgtccat*; reverse primer *gcaaattctggctggtcagt*). An aberrant product (*) is seen in the affected person at 614bp, whereas this product is not found in controls. B) Schematic representation of normal and aberrant product for M1. C) Sequence analysis of the normal sized cDNA product shows the transition from exon 2 to 3. D) Sequence analysis of the larger sized cDNA product shows the transition from exon 2 to intron 2 spanning the deleted 6bp. E) Sequence analysis of the larger sized cDNA product shows the transition from intron 2 to exon 3 at the position of c.49+483. F) Sequence analysis of genomic DNA product of an affected individual shows the transition from exon 2 to intron 2 spanning the deletion of 6bp. G) Sequence analysis of genomic DNA product of a carrier (parent) shows the transition from exon 2 to intron 2 spanning the deletion of 6bp.

Figure 2

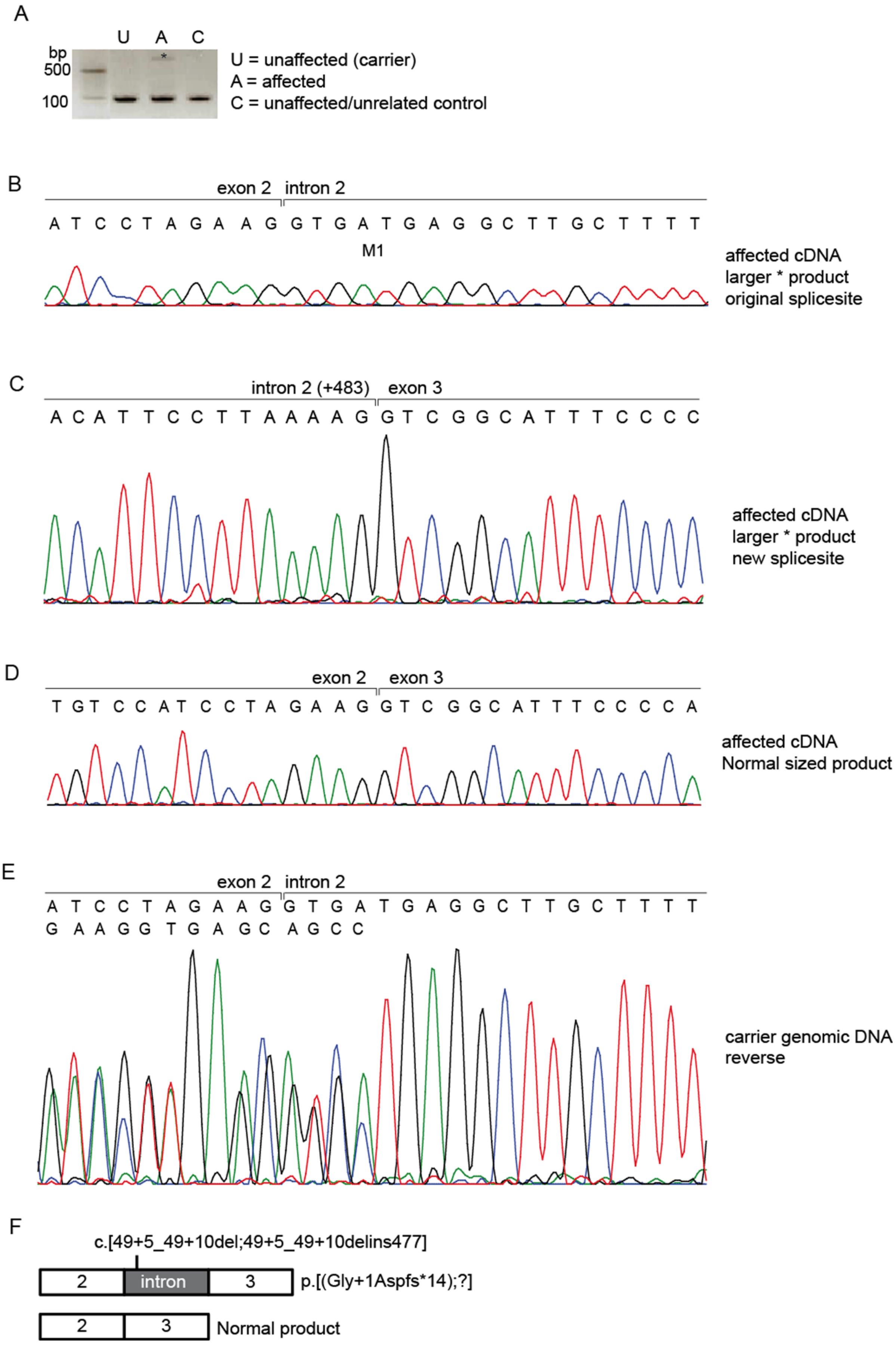

A
U A C
bp
500
100
*
U = unaffected (carrier)
A = affected
C = unaffected/unrelated control
B
exon 2 intron 2
A T C C T A G A A G G T G A T G A G G C T T G C T T T T
M1
affected cDNA
larger * product
original splicesite
C
intron 2 (+483) exon 3
A C A T T C C T T A A A A G G T C G G C A T T T C C C C
affected cDNA
larger * product
new splicesite
D
exon 2 exon 3
T G T C C A T C C T A G A A G G T C G G C A T T T C C C C A
affected cDNA
Normal sized product
E
exon 2 intron 2
A T C C T A G A A G G T G A T G A G G C T T G C T T T T
G A A G G T G A G C A G C C
carrier genomic DNA
reverse
F
c.[49+5_49+10del;49+5_49+10delins477]
2 intron 3
p.[(Gly+1Aspfs*14);?]
2 3
Normal product
